# Supplementary figures and images for: INSPIRE: Intensity and spatial information-based deformable image registration
Source: PLoS One. 2023 Mar 3;18(3):e0282432. doi: 10.1371/journal.pone.0282432 (PMC9983883; doi:10.1371/journal.pone.0282432)

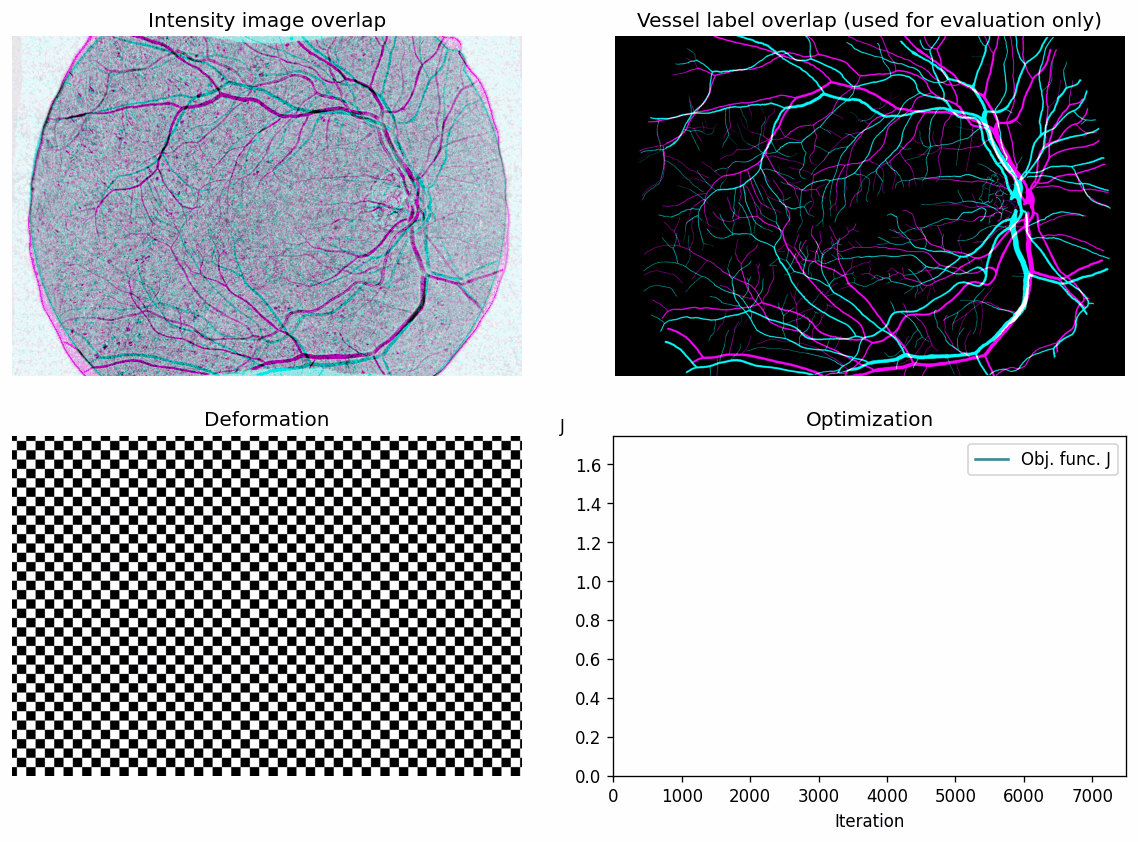

Supplement: S1 File — Magenta/cyan representations of the intensity images (the better the alignment, the less magenta/cyan colour is visible in the image), binary vessel masks (only for evaluation purposes, not used for registration), and the deformation field displayed as a chessboard pattern are included, as well as the objective function J of the registration plotted as a function of the iteration number. (GIF) [file pone.0282432.s001.gif]
