# Supplementary material for: INSPIRE: Intensity and spatial information-based deformable image registration
Source: PLoS One. 2023 Mar 3;18(3):e0282432. doi: 10.1371/journal.pone.0282432 (PMC9983883; doi:10.1371/journal.pone.0282432)
Supplement: Attachment — Submitted filename: Response to Reviewers.pdf [file pone.0282432.s002.pdf]

Respected Dr. Raja, and reviewers 1 and 2,

We thank you for your comments and suggestions regarding the submitted manuscript PONE-D-22-32958 (INSPIRE: Intensity and spatial information-based deformable image registration).

Here we respond point-by-point to the concerns and suggestions raised. The added text in the manuscript has been annotated by a blue font in the version of the manuscript that tracks changes.

Points raised by the editor

1. Please ensure that your manuscript meets PLOS ONE's style requirements, including those for file naming. The PLOS ONE style templates can be found at https://journals.plos.org/plosone/s/file?id=wjVg/PLOSOne_formatting_sample_main_body.pdf and https://journals.plos.org/plosone/s/file?id=ba62/PLOSOne_formatting_sample_title_authors_affiliations.pdf

Answer to point 1:
We have looked over the instructions and changed the file extension of the figures to ".tif", from ".png" to adhere to the requirements.

2. For studies reporting research involving human participants, PLOS ONE requires authors to confirm that this specific study was reviewed and approved by an institutional review board (ethics committee) before the study began. Please provide the specific name of the ethics committee/IRB that approved your study, or explain why you did not seek approval in this case.

Once you have amended this/these statement(s) in the Methods section of the manuscript, please add the same text to the "Ethics Statement" field of the submission form (via "Edit Submission").

For additional information about PLOS ONE ethical requirements for human subjects research, please refer to http://journals.plos.org/plosone/s/submission-guidelines#loc-human-subjects-research.

3. Please provide additional details regarding participant consent. In the ethics statement in the Methods and online submission information, please ensure that you have specified (1) whether consent was informed and (2) what type you obtained (for instance, written or verbal, and if verbal, how it was documented and witnessed). If your study included minors, state whether you obtained consent from parents or guardians. If the need for consent was waived by the ethics committee, please include this information.

If you are reporting a retrospective study of medical records or archived samples, please ensure that you have discussed whether all data were fully anonymized before you

accessed them and/or whether the IRB or ethics committee waived the requirement for informed consent. If patients provided informed written consent to have data from their medical records used in research, please include this information.

Once you have amended this/these statement(s) in the Methods section of the manuscript, please add the same text to the “Ethics Statement” field of the submission form (via “Edit Submission”).

For additional information about PLOS ONE ethical requirements for human subjects research, please refer to
http://journals.plos.org/plosone/s/submission-guidelines#loc-human-subjects-research.

Answer to point 2 and 3:
The High Resolution Fundus Image Database, which the synthetically deformed dataset is based on, consists of a publicly available and anonymized dataset of retinal images. This is a retrospective study and only anonymized data is used, thus ethical approval is not required.

The FIRE dataset consists of 134 images from 39 male and female patients who provided informed consent for the analysis: ``Written informed consent was obtained before data acquisition and processing.'' Since we only perform a retrospective study on these images for the stated purpose, ethical approval was not required.

The 3D study has been performed retrospectively on four datasets of skull-stripped and anonymized brain volume images used soley to evaluate performance of registration methods, a study which requires no ethical approval.

This statement has been added to the manuscript as 4.4. and added to the Ethics Statement related to the submission.

4. In your Data Availability statement, you have not specified where the minimal data set underlying the results described in your manuscript can be found. PLOS defines a study's minimal data set as the underlying data used to reach the conclusions drawn in the manuscript and any additional data required to replicate the reported study findings in their entirety. All PLOS journals require that the minimal data set be made fully available. For more information about our data policy, please see
http://journals.plos.org/plosone/s/data-availability.

Upon re-submitting your revised manuscript, please upload your study’s minimal underlying data set as either Supporting Information files or to a stable, public repository and include the relevant URLs, DOIs, or accession numbers within your revised cover letter. For a list of acceptable repositories, please see
http://journals.plos.org/plosone/s/data-availability#loc-recommended-repositories. Any potentially identifying patient information must be fully anonymized.

Important: If there are ethical or legal restrictions to sharing your data publicly, please explain these restrictions in detail. Please see our guidelines for more information on what we consider unacceptable restrictions to publicly sharing data:

http://journals.plos.org/plosone/s/data-availability#loc-unacceptable-data-access-restrictions. Note that it is not acceptable for the authors to be the sole named individuals responsible for ensuring data access.

We will update your Data Availability statement to reflect the information you provide in your cover letter.

Answer to point 4:
The minimal dataset can be found at https://doi.org/10.5281/zenodo.7552884 which we now reference in the manuscript.

Points related to qualitative results

Reviewer 1:
There is no visual demonstration of the method. Of course the quantitative evaluation of the approach is a significant and valuable benchmark. Nevertheless, it does not provide a qualitative demonstration of the effect of the deformable transformation in the resultant images. The images provided in Figures 1 & 2 are rather unclear and do not suffice for such a demonstration.

Answer:
Thank you for raising this point. In response to this concern, we have included an additional supplementary animation media file as S1, which displays a full registration process of a pair of synthetically deformed retinal images, including both the intensity images overlapping, the vessel masks overlapping (only for evaluation purposes) and a deformed chessboard pattern which illustrates how the space is deformed. We also include a plot where the value of the objective function is plotted as a function of the iteration number. We hope that this will provide more insight into how the method operates and what the final deformations are like.

Points related to the FIRE dataset

Reviewer 1:
In the experiments with the retinal images, authors had to resort into synthetically deforming the images, in order to simulate deformable distortions. However, open datasets for registration of retinal images, that exhibit these properties, already exist (e.g. the FIRE dataset).

Reviewer 2:
Evaluation section does an adequate job, providing metrics and comparisons both for 2D and 3D registrations. My biggest gripe here is that for the 2D registration, the authors perform registration of retinal images, but they create their own synthetic dataset instead of utilizing the publicly available FIRE dataset, which is widely utilized in this domain and that would also allow for easy comparison with domain specific registration methods, not only with general methods. Analysis of performance for 3D registration on brain MRI scans i good.

Answer:
Thank you both for raising this very relevant point. The reason that we used a synthetically deformed dataset was to enable benchmarking of only the deformable component of the transformation, without an affine component which different methods may resolve in different ways. This also enabled use of a very detailed ground-truth mask for an equally detailed evaluation. It is, however, very useful to also evaluate our method on this real dataset (FIRE), constituting a more realistic setup. In our revised version of the manuscript, we have included an evaluation of the proposed method (using a few preprocessing steps to transform the images into a suitable form for INSPIRE) on the FIRE dataset, yielding very good performance. We compare INSPIRE to several existing (domain-specific methods) and conclude that INSPIRE outperforms them all. This part of the empirical study has been added as a new subsection in Section 4. The abstract, introduction, conclusion and the reference list has also changed as a consequence of these changes.

We hope that these answers and changes to the manuscript answers all your concerns to your satisfaction.

With best regards,
Johan Öfverstedt, Joakim Lindblad, and Nataša Sladoje
